# Predictive mapping of the global power system using open data

**DOI:** 10.1038/s41597-019-0347-4

**Published:** 2020-01-15

**Authors:** C. Arderne, C. Zorn, C. Nicolas, E. E. Koks

**Affiliations:** 1 World Bank Group, Washington, D.C. USA; 2 Environmental Change Institute, University of Oxford, Oxford, UK; 3 Department of Civil and Environmental Engineering, University of Auckland, Auckland, New Zealand; 4 Institute for Environmental Studies, Vrije Universiteit Amsterdam, Amsterdam, The Netherlands

**Keywords:** Energy supply and demand, Developing world, Natural hazards, Industry, Energy access

## Abstract

Limited data on global power infrastructure makes it difficult to respond to challenges in electricity access and climate change. Although high-voltage data on transmission networks are often available, medium- and low-voltage data are often non-existent or unavailable. This presents a challenge for practitioners working on the electricity access agenda, power sector resilience or climate change adaptation. Using state-of-the-art algorithms in geospatial data analysis, we create a first composite map of the global power system with an open license. We find that 97% of the global population lives within 10 km of a MV line, but with large variations between regions and income levels. We show an accuracy of 75% across our validation set of 14 countries, and we demonstrate the value of these data at both a national and regional level. The results from this study pave the way for improved efforts in electricity modelling and planning and are an important step in tackling the Sustainable Development Goals.

## Abstract (summary)

**Table:** 

Measurement(s)	electric power system • public utility line
Technology Type(s)	digital curation • computational modeling technique
Factor Type(s)	geographic location
Sample Characteristic - Location	Earth (planet)

**Machine-accessible metadata file describing the reported data:** 10.6084/m9.figshare.11298584

## Background & Summary

Reliable infrastructure networks form the backbone of a prosperous modern economy, with electricity networks providing a central role in helping to deliver health, education, and other infrastructure services. However, 11% of the global population still lacks access to a reliable electricity supply^1^ with lower income countries disproportionately represented. Burundi, for instance, still has over 90% of the population without electricity access. In response, Sustainable Development Goal 7 (SDG 7) calls for universal access to affordable, reliable, and modern energy services by 2030^2^. Achieving this goal is similarly important in achieving other SDGs^3–5^ and as such requires a coordinated effort – not only in the targeted expansion of electricity networks, but also towards increases in renewable energy share of the global energy mix and improved network resilience. To measure the joint global progress on achieving SDG 7, detailed spatial information on the current locations and properties of electricity infrastructure is essential.

Physical assets, such as generation plants, transmission networks (typically higher voltage lines used for the bulk movement of electricity), distribution networks (typically lower voltage lines for distributing electricity from transmission networks to end consumers), substations (for transforming between voltages), and related assets are important to assess the practicalities of connecting isolated communities to wider transmission networks. While more developed countries and their infrastructure owners/operators could be expected to hold suitably complete inventories and geospatial datasets, many lower-income countries data on infrastructure and planned investments are often outdated or incomplete^6^. This makes it difficult for government bodies and utility companies to plan for extending, strengthening, and modifying their electricity networks. Even in advanced economies, where this network data may exist, it can be difficult to find it in an open and standardized way. Projects such as OpenStreetMap have greatly democratized access to geospatial data generally^7^, and while there have been significant efforts on collating data on electricity generation infrastructure^8^, and high voltage transmission lines in OpenGridMap^9^, a globally consistent database of medium and lower voltage distribution networks still lag. Reasons for this can be attributed to the significantly larger number of distribution infrastructure owners/operators required to contribute data (compared to transmission operators which are often national scale bodies), non-existent datasets in GIS-ready formats, restricted public access for security reasons, and the difficulties in digitizing from openly available aerial photographs, whether this be due to the generally smaller overhead line structures or the fact cables are often buried in many urban centers.

In addition to the physical assets, the geospatial locations of electrified settlements within each country is often still unknown. To fill this gap, several studies have developed methods to map these locations and compute accessibility metrics when combined with high-resolution population maps. However, applications have largely been focused on country or sub-continental scales^10–12^. While these methods are showing great promise, a globally consistent approach and map of accessibility is still lacking which can ultimately aid development and planning efforts in attaining SDG 7.

In response, we present the first composite map of the global power grid using publicly available open data – generated through the new open-source tool *gridfinder*^13^, based on work by Rohrer at Facebook: https://code.fb.com/connectivity/electrical-grid-mapping. This tool applies multiple filtering algorithms to night-time light imagery to identify locations most likely to be producing light from electricity. These light sources (target-locations) are then connected to known electricity networks through a least-cost routing algorithm following roads and known distribution lines (adopted from OpenStreetMap). This results in connected networks at two voltage levels we define as high voltage (HV, >70 kV) and medium voltage (MV, 10–70 kV). The dataset shows 97% of the global population reside within 10 km of electricity lines of >10 kV, where the vast majority of those over 10 km being across Sub-Saharan Africa.

The dataset is validated two-fold. Firstly, against 16 electricity networks across 14 countries representing the range of World Bank income groupings: High, Upper-Middle, Lower-Middle, and Low. Across an equal-area grid (edge length 15 km), we compare observed and *gridfinder* delineated networks to show a predictive accuracy of 75%. Secondly, we evaluate the effectiveness of the dataset in predicting accessibility with respect to income groups, human development index (HDI), and investment requirements as commonly reported in the literature and used as key reporting metrics.

A further accompanying dataset is presented representing the density of low voltage (LV) infrastructure, herein defined as below 1 kV. The small geographic scale and prevalence of buried LV cables push the boundaries of satellite imagery detecting abilities. To create this dataset we therefore turn to spatial heuristics to create a weighted layer of electricity access rate by applying an algorithm calibrated to national urban and rural accessibility statistics.

Results of this study pave the way for improved efforts in electricity modelling and planning. Although only predictive, this standardized global dataset of transmission, distribution and low-voltage lines will be a valuable starting point for electrification planners and researchers in several fields, such as assessing social inequalities^14^, estimating exposure to natural hazards^15^, and quantifying electricity infrastructure roll-out requirements^12,16^. An important limitation is that this dataset does not attempt to replicate actual network configurations or precise structures, as would be needed for electrical modelling such as power flow modelling; this was seen as out of scope, and is unlikely to be possible at a global scale with current data sources. The use of open data now allows anyone to freely use or develop this tool (see Code Availability) further to model the power network for any location, as we have demonstrated for the entire globe.

## Methods

**Fig. 1:**
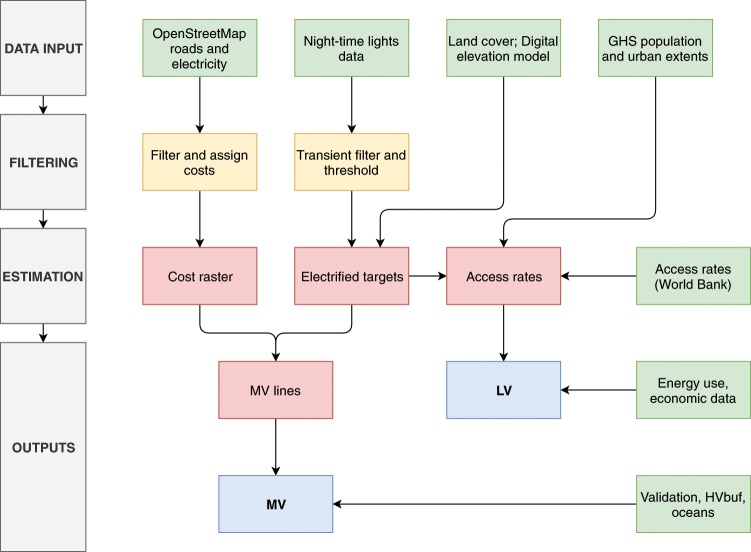
Simplified overview of methodology. Data inputs in green, filtering steps in yellow, intermediate results in red; outputs in blue. The sources of data inputs are either given in Table 1 or provided in the Technical Validation section.

A schematic overview of the computational process for generating a map of the global power grid is illustrated in Fig. 1.

### Input data and processing

The datasets used in generating the global datasets are described in Table 1. To develop the initial high-voltage network layer, there are several existing power network datasets, both open and proprietary. Chief among these is OpenStreetMap, which is relatively comprehensive for transmission lines. Additionally, there are many national and regional datasets released under various licenses, such as SciGRID^17^, and outputs from ENTSO-E, but no attempt at a complete picture of the world. Machine learning methods for feature detection of transmission networks from satellite imagery have made some progress, but still rely heavily on manual tagging^18^. For this exercise, we have determined that the data from OpenStreetMap represent a sufficiently detailed picture of high-voltage transmission lines (here defined as above 70 kV). In some areas, it is comprehensively traced from satellite imagery and ground truth, while in others (particularly developing countries) accuracy and completeness is variable, depending on whether someone has taken the effort to map an area. Although it is often unsuitable for power flow analysis, it is enough for the goal of mapping approximate infrastructure. The vast majority (92%) of the well-tagged data fit our definition of high-voltage, resulting in a 6.6 million km global transmission network.

**Table 1:** Input data sources. All data sources listed are openly available online.

Dataset	Source	Spatial Resolution	Temporal Resolution	Year used
Road/electricity networks	OSM: https://planet.openstreetmap.org	—	daily	2019
Night-time lights	VIIRS-DNB: https://ngdc.noaa.gov/eog/viirs/download_dnb_composites.html	450 m	monthly	2017
Population	GHS-POP: https://ghsl.jrc.ec.europa.eu/ghs_pop2019.php	250 m	annual	2015
Urban extents	GHS-SMOD: https://ghsl.jrc.ec.europa.eu/ghs_smod2019.php	1,000 m	annual	2015
Administrative areas	Natural Earth Data: https://naturalearthdata.com	—	annual	2019
Digital elevation model	NASA SRTM: http://srtm.csi.cgiar.org/srtmdata	1 arc second	annual	2000
Electrification rates	World Bank: https://data.worldbank.org/indicator/EG.ELC.ACCS.ZS	—	annual	2016
Land cover	ESA: https://maps.elie.ucl.ac.be/CCI/viewer	300 m	annual	2015

The goal of this research is to produce a single global dataset that is valuable to researchers and practitioners and easy to replicate, and to serve as a starting point for future improvements and region-specific efforts. This guided the decision to rely exclusively on open data sources such as OpenStreetMap and publicly released satellite imagery and derived products from NASA, NOAA, ESA and others. Focusing on a specific country or region allows the inclusion of locally valuable data sources (e.g. improved grid and road networks, substation locations, improved data on isolated grids, disaggregated access levels), as well as fine-tuning parameters to with attention to local results. In addition, smaller context or additional effort could be used to better understand temporal changes in electricity access and infrastructure.

### Identifying electrification targets

The first step in predicting distribution lines is predicting the points that they connect. The definition of electrification targets as locations likely to be connected to a medium-voltage network informed the approach described below. To filter transient values such as fires and reflections, twelve months of night-time lights imagery from VIIRS^19^ are merged, using for each pixel the 70^th^ percentile value. This value is chosen based on manual testing over several sample countries covering different development levels and land types; higher values (above 80) did not sufficiently filter transient events and extraneous sources (such as fires, gas flaring, snow, and lunar reflections), while lower values (below 60) erased significant sources of real light-emissions. In order to highlight locations that are brighter than their immediate surroundings, thus accentuating even dim points that nonetheless stand out above background light, a two-dimensional filter is convolved over the image with

1 $$F=\left\{\begin{array}{ll}1/{(1+\left|d\right|)}^{3}, & d\ne 0\\ 0, & d=0\end{array}\right.$$

where *d* is that pixel’s perpendicular distance from a given square sample’s centroid. The goal of this filter is to find pixels with a higher value than their neighborhood, biased towards closer areas. To achieve this bias, a non-linear function is needed, and a cubic function was found to achieve better results than a square function. The filter is normalized so that Σ*F* = 0, and the output is then subtracted from the original image.

**Fig. 2:**
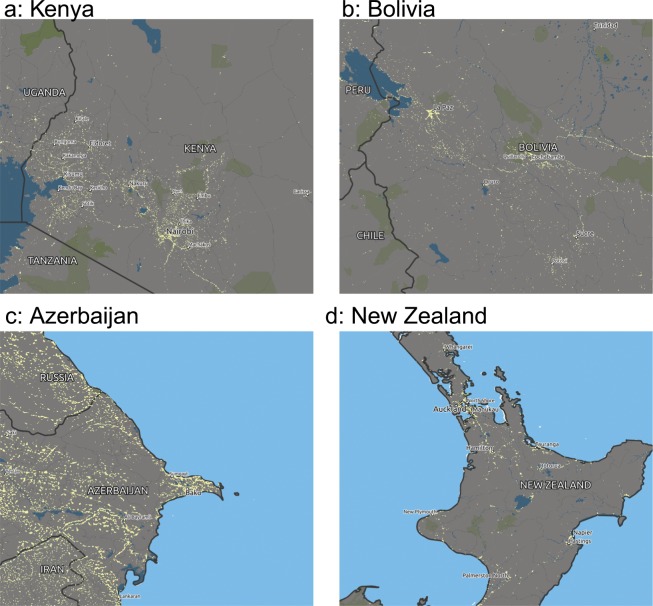
Night-time lights as a proxy for electricity access in four countries with different income levels. (**a**) Kenya, low-income but regions of dense light-emission; (**b**) Bolivia, lower-middle income; (**c**) Azerbaijan, higher-middle income and dense population; (**d**) New Zealand’s North Island, high-income but largely rural. This data can also be visualized interactively here: https://gridfinder.org.

A threshold is then applied to create a binary raster of electrification targets. A more lenient threshold value provides higher granularity in detecting small or marginal locations, but creates many more false positives. As this analysis focuses on distribution-connected locations, we settled on a value of 0.1; lower values (below 0.05) allowed too many false positives (rural areas confirmed via satellite imagery to contain no habitation) while higher values (above 0.2) quickly lost identifiably electrified locations. A different filtering function would require this threshold to be re-examined, and, as above, a closer inspection of a specific geographical areas might reveal a more appropriate location-specific threshold. Additional filters are applied to remove locations with no population, or on slopes above 25° and permanent ice caps, where it very unlikely that distribution lines cross. The output is a global two-dimensional array of target coordinates that must be connected by network lines. An example is given in Fig. 2.

Several studies have validated this approach with ground-truth data^20–22^, but there is yet no standardized methodology. In addition, validations have been done with limited geographical and socio-cultural scope, and with small datasets. Thus, there is significant uncertainty extrapolating these techniques globally. Given most indoor lights do not show up on night-time light imagery; in effect the images measure output from street lights and other large industrial light source. Since our primary purpose is to identify locations very likely connected to the distribution network and not to assess the connection status of individual buildings or households, these issues are of less concern. Attempting to highlight every area with access at a smaller scale would necessitate a closer examination of this issue and more detailed data.

### Deriving MV network distribution lines

To produce data on medium-voltage lines (here defined as 10 to 60 kV), we turn to predictive methods and the tool *gridfinder*^13^. Previous efforts have used Voronoi diagrams and minimum spanning trees, but these require highly detailed data on substations and demand points^9^. The typical approach to a shortest path between points is some form of Dijkstra’s algorithm^23^ (which assumes only two points), while the typical approach to creating a minimum spanning tree is Prim’s algorithm (which assumes a fixed, known distance between points)^24^. Starting with the electrification targets developed above, we apply a many-to-many variant of Dijkstra’s algorithm^23^ with existing road networks as a cost function. The algorithm (see Box 1) greedily adds points as it grows, starting at a single point and branching outwards, keeping track of cost/distance and adding targets as they are found. This process repeats until all cells have been visited.

The algorithm attempts to connect every location with the shortest possible distance, while following roads (preferring larger) where possible. In other words, it attempts to predict distribution networks based on the assumptions of optimum network topology and network lines tending to follow roads. Although both assumptions are frequently false, for a variety of historical reasons, our work indicates that they hold true most of the time: while for cost reasons transmission lines are often built in straight lines, distribution lines generally follow roads making them easier to build and maintain. The algorithm relies exclusively on open and easily accessible datasets (but could be improved with proprietary data sources) and produces medium-voltage network data for any given area and model parameters.

The algorithm is weighted by a cost function based on existing roads from OpenStreetMap. Existing grid lines from OSM are assigned a cost of 0.00; the largest roads (motorways) are assigned a cost of 0.10, the smaller roads (tertiary) a cost of 0.17, and the others in between; these values are arbitrary but achieved a reasonable strategy of preferring larger roads. Areas with no road are assigned a cost of 1.00. This creates a two-dimensional array representing the cost of traversing each cell between the target points. The algorithm in Box 1 is then applied, using the electrified targets and this cost array as inputs. The result is post-processed by removing lines that replicate existing OSM lines, so that the result only shows additions to the OSM data. The final result is therefore disaggregated between lines directly modelled from OSM data, and new lines discovered by the algorithm. These are run individually for each country, under the assumption that MV lines typically do not cross international borders, and then merged into a single global raster. The output is an estimate for each cell of whether it contains a medium-voltage line. To remove predicted lines in remote areas that are unlikely to be connected to the main network, we used a buffer of 100 km on the OSM data and removed all lines beyond that. There was a similar process for predicted submarine lines, for example in Indonesia. Moreover, several administrative areas had to be split or removed, such as exclaves, foreign island territories, or spread-out islands. In addition, some very small city-states and islands were removed, as the algorithm does not yet function well below a certain scale.

**Fig. 3:**
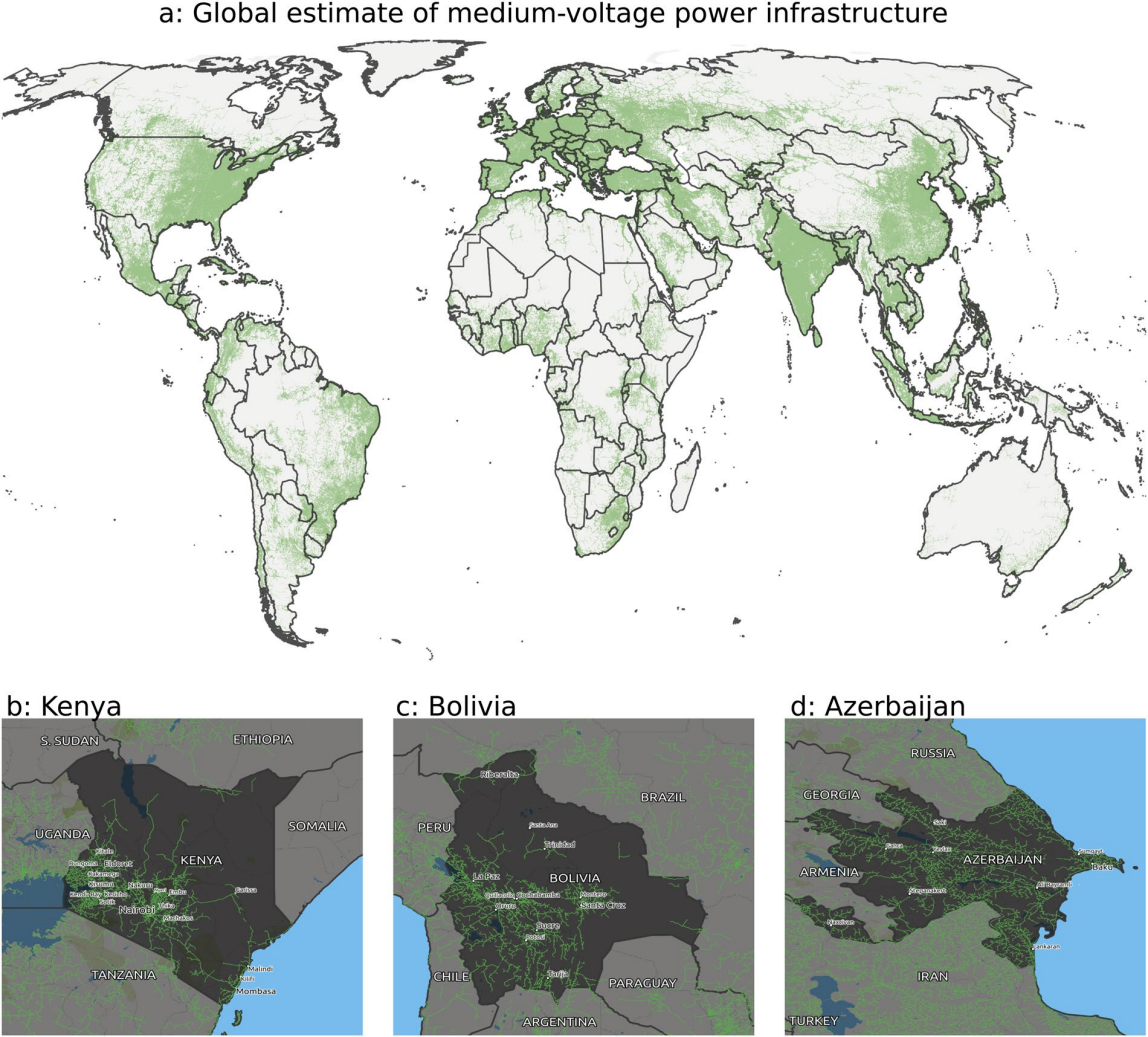
Results of the distribution line modelling. (**a**) Global visualization of predicted distribution lines **(b)** Kenya; **(c)** Bolivia; **(d)** Azerbaijan.

The algorithm is shown in operation for Uganda at https://gridfinder.org/animated. The wider resulting dataset is given in Fig. 3 at the global scale, comprises 7 million km of distribution lines (Fig. 3a) and national/regional scales (Fig. 3b–d).

**Fig. 4:**
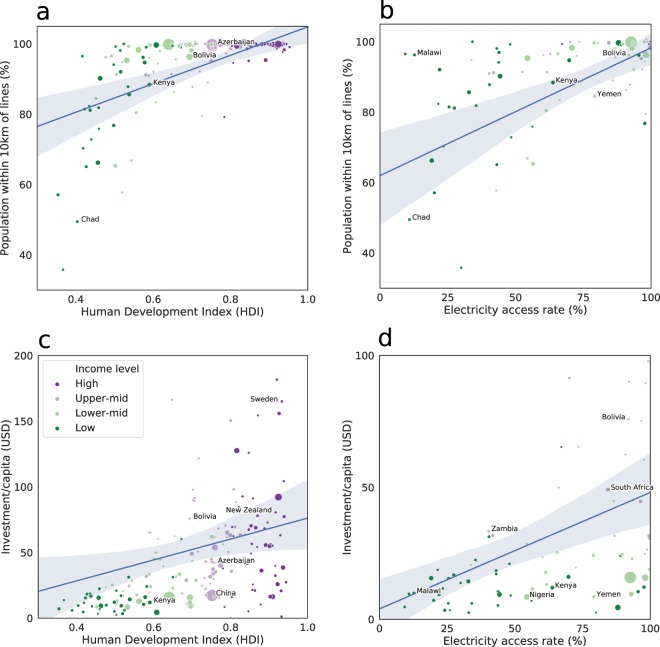
Regional electrical infrastructure proximity and investment value, by country and income level. (**a**) Proportion of population within 10 km of distribution lines by HDI; **(b)** Population within 10 km in low-access countries compared with electricity access rate; **(c)** Infrastructure investment per capita by HDI; **(d)** Infrastructure investment in low-access countries compared with electricity access rate. See Table 1 for data sources with additional data collated for HDI: https://gateway.euro.who.int/en/indicators/hfa_42-0500-undp-human-development-index-hdi.

Overall, *gridfinder* predicts that 97% of the global population lives within 10 km of a MV line, however, large variations are observed between regions, income levels, and across the human development index (HDI) indicator (Fig. 4a,b). In Sub-Saharan Africa, 6% of the population is further than 10 km from the distribution network, while this is around 1% for all other regions. In contrast, South Asia, where although most of the population is close to the network, 10% of the population does not have access to electricity^1^. This highlights an important issue in energy access — network extension is a necessary but non-sufficient factor of electrification. If the households’ willingness to pay is too low, living close to the network will not change the electrification status of those households. This is an important topic to examine if the Sustainable Development Goals are to be reached.

The investment values (see Fig. 4c,d) are calculated based on the predicted amount of distribution lines, multiplied by a constant cost per kilometer. They show a large discrepancy in resources between different regions and income levels. Geographically spread-out and energy-intensive populations (such as Sweden) have a large amount of infrastructure, while more compact countries can get by on less. Most income and HDI groups are represented below the 50 USD/capita line, indicating that this is not a hard limit to development. These regional differences are likely even bigger since the *gridfinder* tool only connects locations with a single line when, there will frequently be multiple lines, either on the same or different towers to provide redundancy in the network. Therefore, these investment values may significantly under predict the divergence, as more developed areas, particularly those at greater risk to natural hazards could be more likely to have such redundancies built in.

### Local access heuristics

In countries with universal access to electricity, accessibility maps mirror population distribution. However, in countries with lower access rates, we turn to night-time lights, urban extents, the output of our *gridfinder* analysis, and national statistics to attempt to quantify the number of people in each grid cell with meaningful electricity access. We apply an iterative algorithm (outlined in Box 2) to produce a gridded surface representing the number of people estimated to have electricity access in each cell. The goal of this algorithm is to match national statistics on urban and rural access rates, and to heuristically apply these rates based on population density, overall brightness, and brightness per capita. This algorithm works by splitting each country into eight groups with the urban-rural split on one dimension and four quantiles of population density on the other. We therefore aim to capture variations in brightness per capita between and within each of these groups. This process is iterated, modifying the factor connecting brightness and access for each group, until a result is achieved satisfactorily close to national statistics. This is limited by being based purely on national statistics (as opposed to sub-nationally disaggregated data), and subsequent research for specific countries or regions could improve on this.

**Fig. 5:**
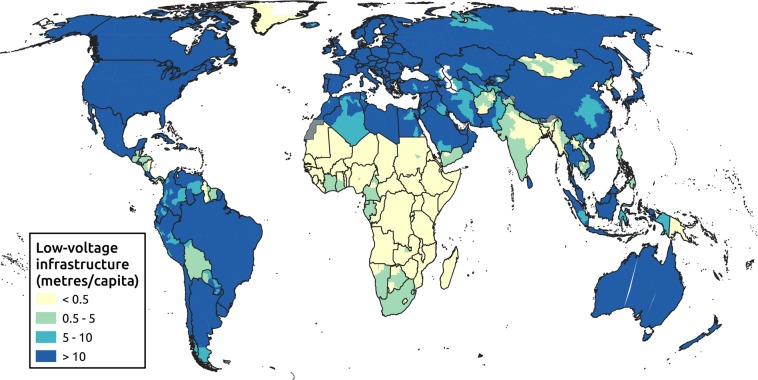
Choropleth of predicted global low-voltage infrastructure per capita. Map shows the level of installed low-voltage lines, disaggregated into four levels.

The resulting geospatial database is used to estimate the length of lines in each cell based on population, demand and household size^16^, according to:

2 $$length=\frac{demand\sqrt{area\times hh\times pop}}{capacity}$$

where *length* is the length of low-voltage lines in that cell, *demand* is the peak per capita demand, *area* is the cell size, *hh* is the number of people per household, *pop* is the number of electrified people in that cell (as defined in Box 1), and *capacity* is the power capacity of the low-voltage line. This equation is derived from a consideration of the number of lines needed to serve a given capacity, as well as the physical length needed to reach all buildings in an area^17^. Parameters were varied by region and calibrate against detailed low-voltage network data. These are then combined to create a single global raster of low-voltage infrastructure comprising 69 million km that can be used to estimate investment costs, and combined with the access estimate to calculate connection costs per household. Results are presented in Fig. 5, highlighting the disparities between low- and high-access countries.

Box 1. Simplified algorithm for connecting electrification targets. The complete code is available in the code repository (see Code Availability, where the earlier Rohrer algorithm is also referenced). An example showing how this works in practice is also available: https://gridfinder.org/algo Parameters:

1. **targets**: binary array of locations with electrified equal to 1
2. **costs**: array of same shape with values [0, 1] defining cost of traversing

Procedure:

1. **dist** = array of same shape as targets storing distance to last electrified
2. **queue = **heap of locations sorted by distance to grid
3. **start = **first location with **targets ==** 1
4. add start to **queue**
5. *while* length(**queue**) > 0:
6.       **current = **top location from **queue**
7.       *for each* location around current:
8.             *if* **location** is connected:
9.                 set **current** as parent of **location**
10.                 follow back from **location** setting all as grid
11.             *else*:
12.                 calculate distance from previous
13. *                if* **location** already visited and distance < previous distance:
14.                       change distance for **location**
15.                 set **location** as visited
16.                 add location to queue

Box 2. Algorithm for estimating population with access to electricity from night light data. The complete code is available online in the code repository (see Code Availability) Parameters:

1. **pop**: 2D array of population
2. **urban**: same-shape binary array of urban/rural
3. **ntl**: same-shape array with night-time lights values
4. **targets**: same-shape binary array showing whether electrified
5. **access**: access rates disaggregated into total, urban, rural
6. **n**: percentile for weights calculation

Procedure:

1. *if* access.urban >90%:
2.       pop_access = pop × access.urban + pop × access.rural3.
3. else:
4.       remove all >1 km from **targets**
5.       *for each* **level** in **urban**:
6.           *for each* **quartile** of **pop**:
7.                  get **pop** at this **level** and **quartile**
8.                  **B = ** brightness per person for each cell
9.                  **b = ** brightness at **n**th percentile of group
10.                  **W =** 1 − (**b** − **B**)/**B**
11.       combine all **W**
12. *      pop_access = W × pop14.*
13.       *if* abs(pop_**access** – **access**.**total**) > 2%:
14.           adjust **n** and repeat from line 1

## Data Records

All resulting data are hosted online^25^ at the following 10.5281/zenodo.3538890 in both vector and raster formats and is freely available under a CC BY 4.0 license. The data are further visualized at https://gridfinder.org. All datasets used in the study are either cited in the Methods section or listed in Table 1.

## Technical Validation

### MV network validation

To validate the medium-voltage results, we consider 16 different networks from 14 countries to represent a wide range of electricity network development and accessibility. Datasets were downloaded as open data or under license to ITRC researchers from Enexis (Netherlands); Ergon, and Western Power (Australia); Western Power and Energy NorthWest (UK); GeoBolivia (Bolivia); and Westpower (New Zealand). Remaining validation datasets were supplied by the World Bank Group, some of which are openly available (https://datacatalog.worldbank.org). Countries represented in each income group are: Australia, Netherlands, New Zealand, United Kingdom (High Income), Namibia (Higher Middle Income), Bolivia, Kenya, Nigeria, Zambia (Lower Middle Income), Burundi, Ethiopia, Malawi, Tanzania, and Uganda (Low Income).

Model performance was then measured by comparing the presence of observed lines (from the above country data) to those produced by the *gridfinder* model across a grid of equal area cells fitted to the electricity distribution extent of each validation set. These grid squares ranged from 500 m up to 100 km (Supplementary Fig. 1 compares predictions at varying resolutions). For each cell, the presence of an observed line form the validation set and the predicted presence of an MV line are computed and given a binary classification. Each cell is then assigned a True-Positive (TP), True-Negative (TN), False-Positive (FP) or False-Negative (FN) attribute. A true-positive (TP) reading would be given if both an MV line is observed in the validation dataset and *gridfinder* predicts the presence of a MV line. A True-Negative (TN) reading would be given if both no MV line is observed in the validation dataset and no MV line is predicted by *gridfinder*. A FP reading indicates *gridfinder* predicts an MV line that is not observed, and finally a FN reading indicates the opposite such that *gridfinder* does not predict an MV line that is observed.

We do not attempt to validate the electrical validity or accuracy of the results, as this is out of scope of our objective and unlikely to be possible on a global scale with current data sources. Follow-up work could attempt to verify these aspects for individual countries, using location-specific data on topology and structures. This limitation means these data are less suited for detailed electrical modelling.

Performance metrics considered include (i) precision, (ii) recall, (iii) accuracy, and (iv) intersection-over-union (IOU). Precision (Eq. 3) is a measure of how likely an MV network is observed given that the model has predicted a MV line is in place. The precision metric penalizes *gridfinder* for predicting the presence of a MV line within the cell when there is no line observed. Maximizing precision ensures *gridfinder* is not overestimating the presence of lines and thus providing a false sense of true accessibility. This is therefore a critical metric to consider.

3 $${\rm{Precision}}\,{\rm{(}} \% {\rm{)}}={\rm{100}}\frac{{\rm{TP}}}{{\rm{TP}}+{\rm{FP}}}$$

Accuracy (Eq. 4) is simply a measure of the total correct predictions over the total validation set. This metric provides a good overview but can be misleading should there be large areas where it is comparatively easy to predict an absence of an electricity network – such as over extreme topographies (mountain ranges or deserts) or large forested areas, amongst others. In such instances, the overall accuracy metric is heavily influenced by the large proportion of TN.

4 $${\rm{Accuracy}}\,{\rm{(}} \% {\rm{)}}={\rm{100}}\frac{{\rm{TP}}+{\rm{TN}}}{{\rm{TP}}+{\rm{TN}}+{\rm{FP}}+{\rm{FN}}}$$

IOU is an alternative metric that excludes TN predictions is the IOU (Eq. 5). Adopted from the object detection literature, here the IOU evaluates how well the model predicts the known presence of distribution lines – disregarding any areas where the model and observed data agree there are no MV lines. While a target of 100% is ideal, object detection models assume IOU > 50% as satisfactory and should be a minimum target.

5 $${\rm{IOU}}\,( \% )=100\frac{{\rm{TP}}}{{\rm{TP}}+{\rm{FN}}+{\rm{FP}}}$$

where all of these metrics in Eqs. 2–4 range between 0–100% with 100% being the most desirable.

**Fig. 6:**
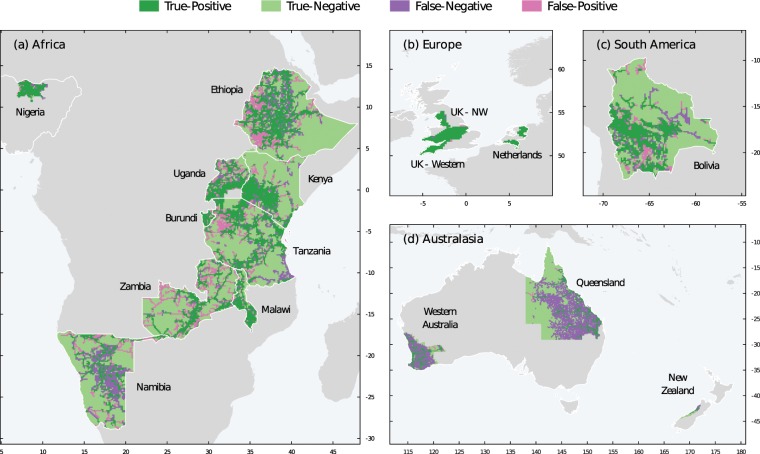
Gridfinder validation at 15 km grid resolution with confusion matrix categories across continents. Each panel represents the countries across different regions namely: (**a**) Africa; (**b**) Europe; (**c**) South America; (**d**) Australasia.

Supplementary Fig. 1 tracks these metrics for a range of grid sizes. Under the above assumption of IOU > 50% being a target, a grid size of 15 km is suggested most appropriate for viewing the dataset (Supplementary Table 1). At this scale, the validation dataset comprises 34800 individual cells with *gridfinder* predicting the following readings: TP: 8832 (25% of dataset), TN: 17270 (50%), FN: 5938 (17%), and FP: 2760 (8%). These results are shown spatially in Fig. 6.

Overall, we observe validation performance not necessarily correlating directly with country income group. While all networks show validation precision of >50% (Supplementary Table 1, Supplementary Figs. 2 and 4) lower accuracies are observed for Australia (both Queensland and Western Australia). We attribute this to relatively low night light in rural areas such that *gridfinder* does not have as many target electrification sites, however, given accessibility is in-fact 100%, one would expect more MV lines to be present across the distribution extent. While this result may be concerning for predicting accessibility in geographies comparable to Australia, the already 100% access and highly detailed geospatial datasets (often openly available) mean the *gridfinder* is not necessarily required in such areas – with much more focus and application expected in those regions targeting SDG7.

In these regions targeting SDG7 and which *gridfinder* is of the greatest initial interest, the main inconsistencies in these results stem from connected villages that fail to show up in the filtered data, due to not producing enough light above background noise. In many cases, these villages will be technically connected but very few households will have electricity access. Incorrect routing assumptions, where distribution lines do not follow a road, or try to avoid a topographical feature, which is not currently accounted for, also produce mistakes. Finally, as the algorithm produces a minimum spanning tree, there are no predicted redundant lines, which are frequently built for reliability reasons.

This type of experimental work presents a difficult conundrum: we are developing new data with no precedent, so there is a limit to the validation that is possible, and therefore the ability to iterate and improve results is severely limited. For example, the results could be improved by using daily, rather than monthly, satellite imagery, in order to finely control the filtering process. However, optimizing this process will require highly detailed ground-truth data, which are only available in limited areas. Further, strong geographic variations limit the ability to generalize findings based on these data. These findings should be viewed as a step forwards, and an enabler of new analysis and additional ground-truthing; they should not be relied upon for decision-making without independent validation.

### Local access validation

Overall, our results compare favorably with recent work in the literature, which is performed at regional scales^11,26^. However, at the global scale, further calibration will need to be sought over time as more datasets become available. In urban areas, we find an over prediction of access rates, whereas the algorithm under predicts in rural areas. This over prediction is primarily caused by bright industry and streetlights, which do not necessarily imply household connections. Conversely, rural villages may have access that provides valuable services, even if weak or intermittent, but may not produce enough light to rise above the noise. In most cases, it was possible to calibrate the results to closely match official World Bank statistics (see Supplementary Fig. 5). However, in several cases it was not possible, and the model results are significantly off the World Bank statistics. These cases highlighted limitations with our modelling approach, but also surfaced interesting realities about the distribution of electricity access between urban and rural areas. The clearest failures were Pakistan and Libya, both with modelled access rates close to 100%, but with official rates around 70%. In Pakistan, the 100% official urban access rate is automatically assigned to urban areas. In this case, the limited quality of the population and urban extents, along with differing definitions of urban, means that this already over predicts the actual electricity access rate. In Libya, falling access rates over the last 20 years create a difficult situation for an automated algorithm to handle. On the other hand, the model sometimes predicts access rate lower than the official statistics. It is mostly the case for countries where the reliability of the grid is low and/or where off-grid access is high. Indeed, the World Bank statistics capture both on-grid and off-grid access under the same access rate while our model computes on-grid access only. For example, in Myanmar and Ethiopia, where the World Bank access rates are 70% and 44%, respectively, the model underpredicts rates of 50% and 35% respectively. These are much closer to the official on-grid access rates of 40% and 33% respectively. Unfortunately, this comparison is only possible for a few countries for which the World Bank statistics were established using the MTF survey^27,28^.

## Usage Notes

Datasets are available in standard formats (GeoTIFF for raster data and GeoPackage for vector data) in the WGS84 (EPSG:4326) coordinate system. All data can be viewed and analyzed without any further edits – as shown on gridfinder.org, and are available for download^25^ at the following, 10.5281/zenodo.3538890. The HV and MV data are shared as a single vector data file in the standard GeoPackage format and in the WGS84 coordinate system (EPSG:4326). The LV data are shared as raster data in the standard GeoTIFF format in the World Mollweide projected coordinate system (EPSG:54009), while the electrified targets data are also in raster GeoTIFF format but in the WGS84 coordinate system (EPSG:4326). Access, investment, and total line length results are aggregated to the country level in Supplementary Table 2.

As raised above, the dataset has the potential to be used in a wide range of applications. It will be a valuable starting point for electrification planners who have called for further application of this methodology^11,12^. Similarly, others assessing social inequalities^14^ and estimating exposure to natural hazards^15^ will be able to apply such datasets. With the code easily accessible via GitHub repositories (as given in the Code Availability section), users can apply their own parameters that could better represent their region of interest. Similarly, as more NTL data are published at higher resolutions and continuous additions are being made to OpenStreetMap these datasets can be easily updated with little delay.

## Supplementary information

Supplementary Information

## Data Availability

All scripts used are available at https://github.com/carderne/predictive-mapping-global-power (scripts and routines for preparing and finalizing data), https://github.com/carderne/gridfinder (primary code for preparing night-time lights and creating medium-voltage predictions), and https://github.com/carderne/access-estimator (access estimations and low-voltage predictions). The output data are available at the following, 10.5281/zenodo.3538890. See also the original Facebook algorithm here: https://github.com/facebookresearch/many-to-many-dijkstra.
