# Supplementary material for: Predictive mapping of the global power system using open data
Source: Sci Data. 2020 Jan 15;7:19. doi: 10.1038/s41597-019-0347-4 (PMC6962213; doi:10.1038/s41597-019-0347-4)
Supplement: Supplementary file 1 — Supplementary Information [file 41597_2019_347_MOESM1_ESM.pdf]

Supplementary Information for:

Predictive mapping of the global power system using open data

Authors
C. J. Arderne[1], C. Zorn[2,3], C. Nicolas[1], E.E. Koks[2,4]

Affiliations
1. World Bank Group, Washington, D.C., USA
2. Environmental Change Institute, University of Oxford, Oxford, UK
3. Dept. Of Civil and Environmental Engineering, University of Auckland, Auckland, New Zealand
4. Vrije Universiteit Amsterdam, Amsterdam, The Netherlands

Corresponding author(s): Chris Arderne (carderne@worldbank.org)

Table of Contents

Supplementary Figure 1. Examples varying grid resolution for validation. Examples are shown for Bolivia across multiple resolutions……2

Supplementary Figure 2. Analysis of accuracy, precision, and IOU across the validation dataset. Bold lines indicate the combined global results……3

Supplementary Table 1. Validation Results by country. Grid resolution, accuracy percentage, and precision values all rounded to nearest integer……4

Supplementary Figure 3. Confusion matrix table with collated results across the complete validation set with a validation grid resolution of 15 km……5

Supplementary Figure 4. Validation performance across accuracy and precision metrics. Marker color represents World Bank income group and size represents World Bank estimated population with electricity access……5

Supplementary Figure 5. Modelled electricity access rates compared with World Bank statistics……6

Supplementary Table 2. Raw summary of results aggregated to country levels……7

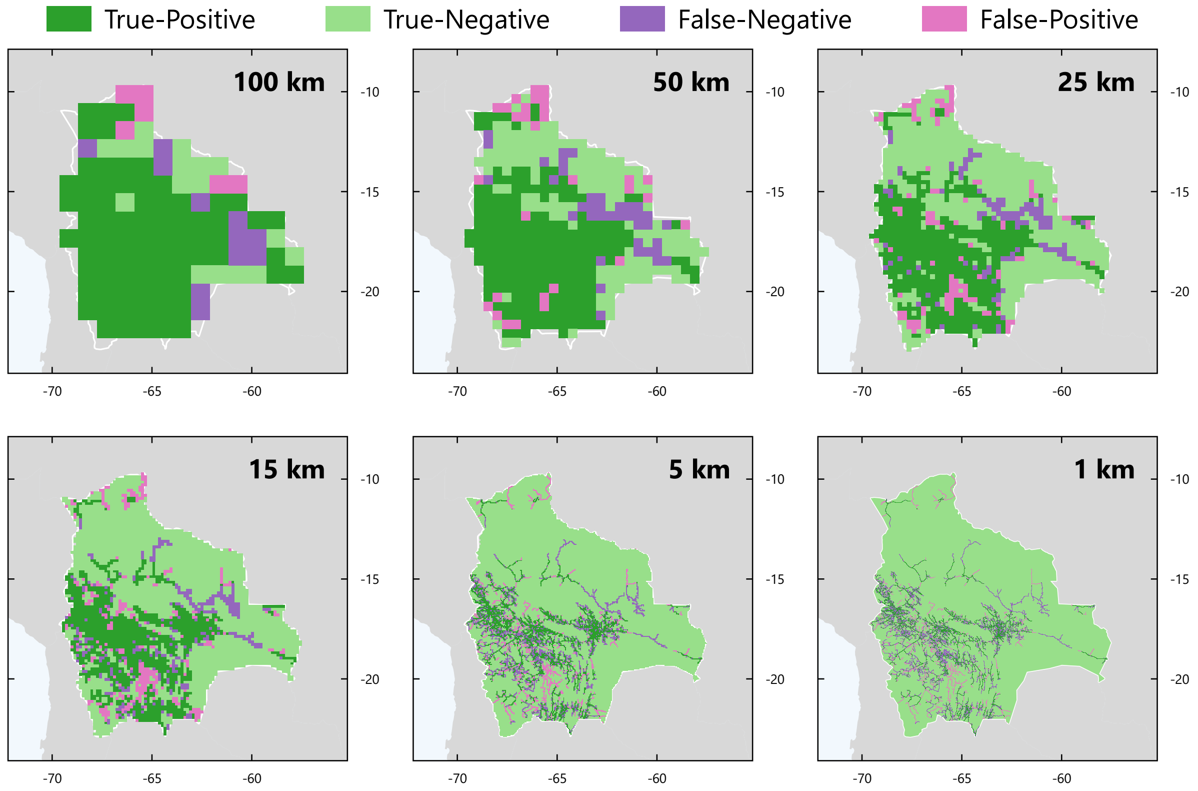

Supplementary Figure 1. Examples varying grid resolution for validation. Examples are shown for Bolivia across multiple resolutions. We see the effect that larger grid sizes (i.e. 100 km) show better predictive capacity, but do not represent the network necessarily well compared to those ≤15 km which better reveal the networks true topology.

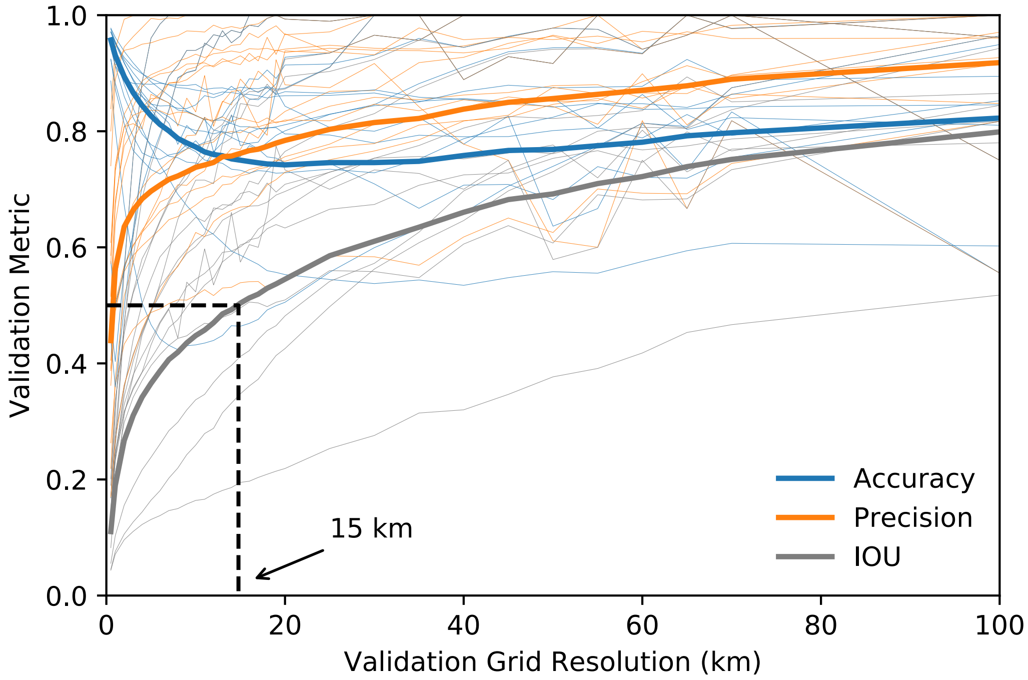

Supplementary Figure 2. Analysis of accuracy, precision, and IOU across the validation dataset. Bold lines indicate the combined global results. Finding the grid resolution of which we can report results with most confidence will depend on the accuracy, precision, and IOU metrics highlighted in the Technical Validation. Median and mean are not considered as this would equally weight each network with potentially misleading results given some datasets are considerably smaller than others (particularly the High Income group countries. When combining all validation grids, a 15 km grid resolution is required to attain IOU ≥ 0.5 (dotted line). Therefore, on the whole, *gridfinder* performs better (or the same) for all Low and Lower-Middle income group countries if the 15 km validation grid is assumed (in 8 out of 9 countries). This is similarly reflected in the accuracy and precision metrics for the global validation resolution. At the 15 km resolution, High and Upper-Middle income group countries are all expected to underperform with 3 out of 6 countries requiring > 22 km validation grids to reach IOU≥0.5.

Supplementary Table 1. Validation Results by country. Grid resolution, accuracy percentage, and precision values all rounded to nearest integer. Accuracy is highest for the largest grid sizes as expected due to the significant increase in (and ease of) TN predictions. In comparison, precision and IOU both increase as the grid cell sizes increase. The IOU has the greatest variability with the lower end representing Queensland (Australia) where *gridfinder* under predicts the presence of MV lines. With the exception of Zambia, all Low Income and Lower-Middle countries reach IOU≥0.5 for resolutions ≤9km.

Country	Income Group	Electricity Access (%)	Resolution for IOU≥0.5	Accuracy at this resolution	Precision at this resolution	Accuracy at 15 km	Precision at 15 km
Australia (WA)	High	100%	**25 km**	56%	94%	46%	93%
Australia (QLD)	High	100%	**90 km**	60%	96%	59%	94%
Bolivia	Lower Middle	91.8%	5 km	89%	73%	86%	80%
Burundi	Low	9.3%	5 km	73%	67%	86%	89%
Ethiopia	Low	44.3%	9 km	81%	66%	80%	72%
Kenya	Lower Middle	63.8%	4 km	88%	83%	88%	82%
Malawi	Low	12.7%	3 km	88%	79%	87%	91%
Namibia	Upper Middle	52.5%	**22 km**	73%	74%	73%	67%
Netherlands	High	100%	2 km	53%	98%	100%	100%
New Zealand	High	100%	9 km	80%	84%	78%	87%
Nigeria	Lower Middle	54.4%	3 km	80%	77%	86%	95%
Tanzania	Low	32.8%	7 km	84%	68%	81%	73%
Uganda	Low	22.0%	5 km	78%	66%	76%	77%
UK (NW)	High	100%	2 km	58%	95%	96%	100%
UK (Western)	High	100%	2 km	53%	94%	97%	98%
Zambia	Lower Middle	40.3%	15 km	78%	54%	78%	54%
**Global**		**46.98%**	**15 km**	**75%**	**76%**	**75%**	**76%**

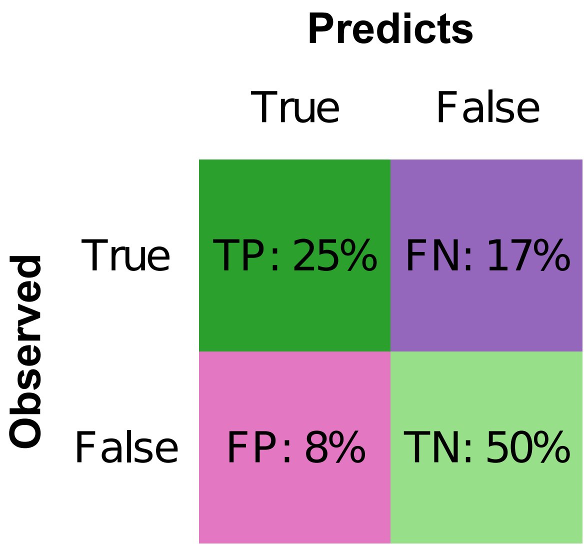

Supplementary Figure 3. Confusion matrix table with collated results across the complete validation set with a validation grid resolution of 15 km. At this scale, approximately 27% of data are located in High Income countries, 10% in Upper-Middle Income, 32% in Lower-Middle Income, and 31% in Low Income countries.

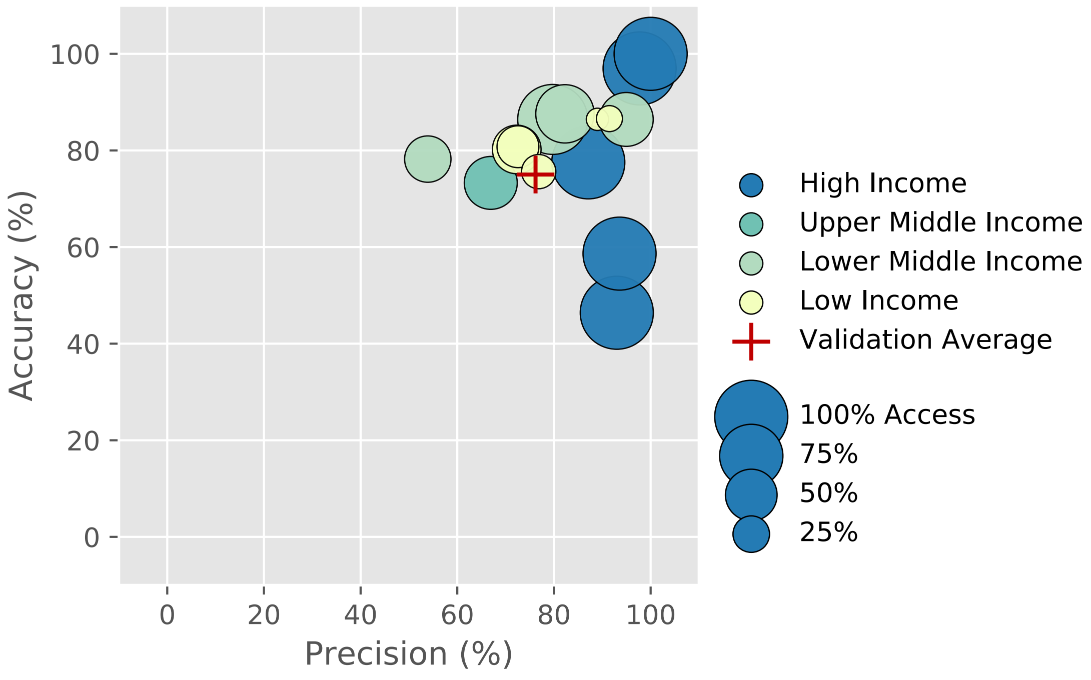

Supplementary Figure 4. Validation performance across accuracy and precision metrics. Marker color represents World Bank income group and size represents World Bank estimated population with electricity access. While all networks show validation precision of >50%, lower accuracies are observed for Australia (both Queensland and Western Australia). Validation performance does not necessarily correlate directly with country income group.

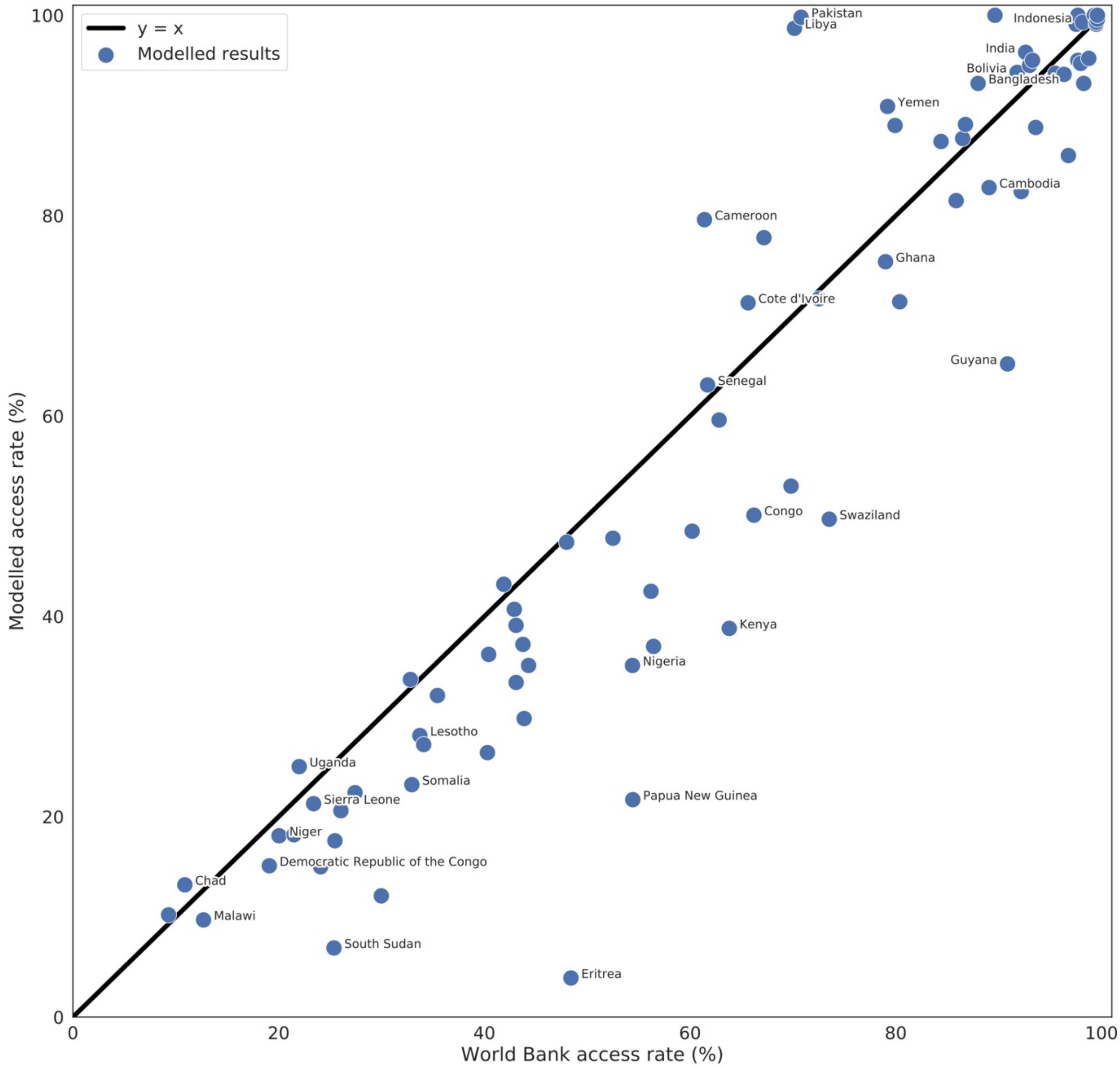

Supplementary Figure 5. Modelled electricity access rates compared with World Bank statistics. There appear to be significant areas of False-Negative predictions (*gridfinder* does not predict the presence of an observed line) in the Australian based validation sets.

Supplementary Table 2. Raw summary of results aggregated to country levels. While each of the high income countries have universal access to electricity, the relationship between accessibility and income groups are not as clear for the remaining countries Upper-Middle, Lower-Middle, and Low Income groupings are not as clear. Bolivia and Zambia being one such example for Lower-Middle income countries with 91.8% and 40.3% electricity access, respectively. This equate to over 50% more of the Bolivian population with electricity access, yet is still classified in the same Income Group as Zambia. Similarly, Namibia (classified as Upper Middle income) has lower accessibility rates than a range of Lower-Middle income groups (Bolivia, Kenya, and Nigeria).

Country	Code	Total MV (km)	Total LV (km)	Population with 10km of MV (%)	MV investment per capita (USD)	Modelled access rate (%)	World Bank access rate (%)
Afghanistan	AFG	13,348	28,069	77%	12	96%	98%
Albania	ALB	4,215	51,793	100%	44	100%	100%
Algeria	DZA	49,430	484,832	99%	37	100%	100%
Andorra	AND	61	1,272	100%	26	97%	100%
Angola	AGO	26,655	6,343	91%	32	43%	42%
Antigua and Barbuda	ATG	116	1,998	100%	38	99%	100%
Argentina	ARG	92,329	590,015	99%	63	100%	100%
Armenia	ARM	3,037	50,060	100%	30	99%	100%
Aruba	ABW	6	2,555	32%	2	100%	100%
Australia	AUS	61,657	544,198	98%	77	100%	100%
Austria	AUT	15,936	227,175	100%	55	100%	100%
Azerbaijan	AZE	13,901	170,807	100%	42	100%	100%
Bahamas	BHS	793	7,873	95%	63	100%	100%
Bahrain	BHR	242	23,737	100%	5	100%	100%
Bangladesh	BGD	24,191	50,586	100%	4	93%	88%
Belarus	BLR	31,188	141,516	100%	98	100%	100%
Belgium	BEL	12,434	294,751	100%	32	100%	100%
Belize	BLZ	1,139	583	101%	89	93%	98%
Benin	BEN	6,354	3,684	98%	17	39%	43%
Bhutan	BTN	1,898	36,577	94%	60	100%	98%
Bolivia	BOL	27,344	10,801	96%	76	94%	92%
Bosnia and Herzegovina	BIH	8,804	66,589	100%	68	100%	100%
Botswana	BWA	9,394	1,739	97%	122	60%	63%
Brazil	BRA	370,664	3,105,837	99%	54	100%	100%
Brunei Darussalam	BRN	699	11,133	100%	49	100%	100%
Bulgaria	BGR	21,083	156,199	100%	88	100%	100%
Burkina Faso	BFA	9,475	2,355	81%	16	18%	25%
Burundi	BDI	1,764	621	97%	5	10%	9%
Cambodia	KHM	12,654	13,454	99%	24	83%	89%
Cameroon	CMR	14,248	14,128	80%	18	80%	61%
Canada	CAN	180,729	713,812	99%	156	100%	100%
Central African Republic	CAF	2,419	387	36%	14	12%	30%
Chad	TCD	4,608	944	49%	10	13%	11%

Country	Code	Total MV (km)	Total LV (km)	Population with 10km of MV (%)	MV investment per capita (USD)	Modelled access rate (%)	World Bank access rate (%)
Chile	CHL	24,889	236,444	99%	42	100%	100%
China	CHN	795,935	17,217,180	100%	17	100%	100%
Colombia	COL	50,765	500,167	98%	32	100%	100%
Comoros	COM	67	445	20%	3	89%	80%
Congo	COG	8,181	1,555	83%	50	50%	66%
Costa Rica	CRI	8,112	79,899	100%	50	100%	100%
Cote d'Ivoire	CIV	19,252	18,379	97%	25	71%	66%
Croatia	HRV	13,415	105,975	100%	93	100%	100%
Cuba	CUB	15,104	146,668	100%	40	100%	100%
Cyprus	CYP	1,524	18,332	100%	54	99%	100%
Czech Republic	CZE	24,201	253,374	100%	68	100%	100%
Democratic Republic of the Congo	COD	40,450	6,692	66%	16	15%	19%
Denmark	DNK	11,927	142,199	100%	63	100%	100%
Djibouti	DJI	694	165	96%	24	49%	60%
Dominica	DMA	123	1,202	100%	51	100%	100%
Dominican Republic	DOM	6,177	127,870	100%	17	100%	100%
Ecuador	ECU	23,173	240,672	100%	43	100%	100%
Egypt	EGY	27,463	851,766	100%	9	100%	100%
El Salvador	SLV	3,892	74,872	100%	19	99%	99%
Equatorial Guinea	GNQ	1,961	684	99%	65	78%	67%
Eritrea	ERI	1,603	158	73%	9	4%	48%
Estonia	EST	6,801	24,803	100%	154	100%	100%
Ethiopia	ETH	31,272	11,130	90%	9	35%	44%
Faroe Islands	FRO	474	3,487	100%	295	100%	100%
Finland	FIN	32,924	101,564	99%	182	100%	100%
France	FRA	118,566	1,607,007	100%	55	100%	100%
Gabon	GAB	5,387	1,932	88%	90	82%	92%
Gambia	GMB	477	666	76%	7	43%	56%
Georgia	GEO	7,546	89,066	100%	56	100%	100%
Germany	DEU	104,401	1,856,241	100%	39	100%	100%
Ghana	GHA	22,350	18,625	100%	24	75%	79%
Greece	GRC	28,570	211,808	100%	78	100%	100%
Guatemala	GTM	12,542	14,366	100%	23	96%	93%
Guinea	GIN	9,509	2,924	82%	22	32%	35%
Guinea-Bissau	GNB	211	269	13%	3	21%	26%
Guyana	GUY	1,602	580	86%	62	65%	91%
Haiti	HTI	1,891	3,699	94%	5	37%	44%
Honduras	HND	12,172	9,822	98%	45	88%	87%
Hungary	HUN	21,037	237,756	100%	64	100%	100%
Iceland	ISL	8,333	5,172	98%	770	96%	100%
India	IND	693,102	604,416	100%	16	96%	93%
Indonesia	IDN	134,709	3,321,484	96%	16	99%	98%

Country	Code	Total MV (km)	Total LV (km)	Population with 10km of MV (%)	MV investment per capita (USD)	Modelled access rate (%)	World Bank access rate (%)
Iran, Islamic Republic of	IRN	167,159	877,169	100%	63	100%	100%
Iraq	IRQ	35,209	377,162	100%	29	100%	100%
Ireland	IRL	16,394	111,745	100%	104	100%	100%
Israel	ISR	4,350	123,292	100%	15	100%	100%
Italy	ITA	72,253	1,293,927	100%	36	100%	100%
Jamaica	JAM	2,847	58,901	100%	31	99%	100%
Japan	JPN	68,597	2,122,796	100%	16	100%	100%
Jordan	JOR	5,683	94,538	100%	22	100%	100%
Kazakhstan	KAZ	89,074	254,192	99%	150	100%	100%
Kenya	KEN	18,577	9,191	88%	12	39%	64%
Korea, Democratic People's Republic of	PRK	8,838	10,913	97%	10	30%	44%
Korea, Republic of	KOR	27,542	525,181	100%	16	100%	100%
Kuwait	KWT	2,113	44,258	100%	16	100%	100%
Kyrgyzstan	KGZ	12,289	76,676	99%	60	98%	100%
Lao People's Democratic Republic	LAO	10,985	12,540	92%	45	89%	94%
Latvia	LVA	8,328	30,678	100%	126	100%	100%
Lebanon	LBN	3,155	74,045	100%	16	100%	100%
Lesotho	LSO	1,485	760	95%	21	28%	34%
Liberia	LBR	2,854	864	82%	19	18%	21%
Libya	LBY	19,130	118,151	100%	91	99%	70%
Liechtenstein	LIE	63	1,516	100%	40	96%	100%
Lithuania	LTU	8,862	48,168	100%	91	100%	100%
Luxembourg	LUX	1,137	15,348	100%	57	100%	100%
Macedonia, the Former Yugoslav Republic of	MKD	4,292	39,260	100%	62	100%	100%
Madagascar	MDG	2,766	1,699	29%	3	15%	24%
Malawi	MWI	5,844	1,974	96%	10	10%	13%
Malaysia	MYS	36,252	561,323	100%	36	100%	100%
Mali	MLI	10,924	3,312	65%	18	33%	43%
Malta	MLT	146	7,493	100%	10	100%	100%
Mauritania	MRT	2,678	2,576	58%	17	41%	43%
Mexico	MEX	170,990	1,666,600	100%	40	100%	100%
Moldova, Republic of	MDA	6,047	101,047	100%	44	100%	100%
Mongolia	MNG	24,657	1,710	89%	249	82%	86%
Montenegro	MNE	1,859	12,328	100%	89	100%	100%
Montserrat	MSR	11	171	100%	61	100%	0%
Morocco	MAR	38,574	410,302	99%	34	100%	100%
Mozambique	MOZ	15,838	4,333	81%	17	22%	27%
Myanmar	MMR	29,026	20,246	95%	16	53%	70%

Country	Code	Total MV (km)	Total LV (km)	Population with 10km of MV (%)	MV investment per capita (USD)	Modelled access rate (%)	World Bank access rate (%)
Namibia	NAM	14,827	1,398	91%	166	48%	53%
Nepal	NPL	10,445	41,525	96%	10	94%	96%
Netherlands	NLD	11,843	381,323	100%	21	100%	100%
New Zealand	NZL	12,168	106,508	99%	81	100%	100%
Nicaragua	NIC	6,429	4,207	96%	32	89%	87%
Niger	NER	4,868	1,801	57%	7	18%	20%
Nigeria	NGA	51,599	40,712	95%	8	35%	54%
Norway	NOR	44,206	120,816	99%	257	100%	100%
Oman	OMN	13,028	48,943	100%	84	99%	100%
Pakistan	PAK	81,767	1,395,834	98%	13	100%	71%
Palestine, State of	PSE	1,858	69,685	99%	11	100%	100%
Panama	PAN	5,796	4,256	97%	44	94%	100%
Papua New Guinea	PNG	7,222	892	67%	29	22%	54%
Paraguay	PRY	22,093	84,680	99%	98	100%	99%
Peru	PER	46,837	360,173	95%	45	94%	96%
Philippines	PHL	31,829	103,710	98%	10	95%	93%
Poland	POL	88,883	809,844	100%	69	100%	100%
Portugal	PRT	23,334	236,687	95%	67	100%	100%
Puerto Rico	PRI	2,355	83,300	100%	19	100%	100%
Qatar	QAT	1,609	23,568	99%	22	100%	100%
Romania	ROU	45,850	468,509	100%	70	100%	100%
Russian Federation	RUS	606,824	2,473,706	99%	128	100%	100%
Rwanda	RWA	2,426	1,973	100%	6	27%	34%
Saint Kitts and Nevis	KNA	83	1,523	100%	45	100%	100%
Saint Lucia	LCA	163	2,930	100%	26	96%	99%
Sao Tome and Principe	STP	125	103	96%	20	72%	73%
Saudi Arabia	SAU	74,038	382,315	99%	70	100%	100%
Senegal	SEN	6,788	4,756	91%	13	63%	62%
Serbia	SRB	16,613	140,709	100%	71	100%	100%
Sierra Leone	SLE	2,540	1,032	70%	12	21%	23%
Singapore	SGP	248	37,077	100%	1	99%	100%
Slovakia	SVK	10,400	138,964	100%	57	100%	100%
Slovenia	SVN	5,078	59,572	100%	73	100%	100%
Somalia	SOM	923	1,440	16%	3	23%	33%
South Africa	ZAF	89,639	58,579	100%	49	87%	84%
South Sudan	SSD	2,513	304	20%	6	7%	25%
Spain	ESP	85,647	808,845	95%	56	100%	100%
Sri Lanka	LKA	11,059	399,763	100%	16	99%	98%
Sudan	SDN	15,509	9,035	65%	12	37%	56%
Suriname	SUR	1,462	1,003	92%	75	86%	97%
Swaziland	SWZ	2,865	806	100%	65	50%	74%
Sweden	SWE	53,622	194,541	100%	165	100%	100%
Switzerland	CHE	7,824	182,703	100%	27	100%	100%

Country	Code	Total MV (km)	Total LV (km)	Population with 10km of MV (%)	MV investment per capita (USD)	Modelled access rate (%)	World Bank access rate (%)
Syrian Arab Republic	SYR	15,302	281,785	99%	25	100%	90%
Taiwan	TWN	6,383	289,720	100%	8	100%	100%
Tajikistan	TJK	9,048	80,315	99%	31	100%	99%
Thailand	THA	78,248	1,086,942	100%	34	100%	100%
Timor-Leste	TLS	1,588	1,873	100%	39	71%	80%
Togo	TGO	5,287	2,458	99%	21	47%	48%
Tonga	TON	88	404	79%	25	95%	98%
Tunisia	TUN	16,196	147,475	100%	43	100%	100%
Turkey	TUR	171,359	1,057,026	100%	65	100%	100%
Turkmenistan	TKM	16,469	53,702	100%	89	97%	100%
Uganda	UGA	12,675	5,152	92%	10	25%	22%
Ukraine	UKR	93,036	1,058,713	100%	62	100%	100%
United Arab Emirates	ARE	6,419	77,177	100%	21	100%	100%
United Kingdom	GBR	55,914	1,065,331	100%	26	100%	100%
United Republic of Tanzania	TZA	25,713	10,088	86%	14	34%	33%
United States	USA	959,811	8,647,420	100%	92	100%	100%
Uruguay	URY	8,107	53,427	99%	69	100%	100%
Uzbekistan	UZB	29,202	451,238	100%	29	100%	100%
Venezuela	VEN	36,953	384,899	99%	35	100%	100%
Viet Nam	VNM	37,526	971,327	100%	12	100%	100%
Yemen	YEM	7,731	18,597	84%	9	91%	79%
Zambia	ZMB	18,605	4,042	91%	33	26%	40%
Zimbabwe	ZWE	16,486	4,812	88%	31	36%	40%
